# Efficacy of a dual task protocol on neurophysiological and clinical outcomes in migraine: a randomized control trial

**DOI:** 10.1007/s10072-024-07611-8

**Published:** 2024-05-29

**Authors:** Manuela Deodato, Antonio Granato, Alex Buoite Stella, Miriam Martini, Enrico Marchetti, Ilaria Lise, Alessandra Galmonte, Luigi Murena, Paolo Manganotti

**Affiliations:** 1 Department of Life Sciences, University of Trieste, Trieste, Italy; 2 Department of Medical, Surgical and Health Sciences, University of Trieste, Trieste, Italy; 3 Azienda Sanitaria Universitaria Giuliano Isontina, Via Pascoli 31, 34100 Trieste, Italy; 4 Azienda Sanitaria Universitaria Giuliano Isontina, Strada Di Fiume 447, 34149 Trieste, Italy

**Keywords:** Episodic migraine, Active exercise, Cognitive training, Dual task protocol, Transcranial magnetic stimulation

## Abstract

The main aim of this study was to investigate the efficacy of a dual task protocol in people with episodic migraine with respect to both active exercises only and cognitive task only treatments, concerning some neurophysiological and clinical outcomes. A randomized control trial was adopted in people with episodic migraine without aura. Some neurophysiological and clinical outcomes were collected (t0): resting motor threshold (rMT), short intracortical inhibition (SICI) and facilitation (ICF), pressure pain threshold (PPT), trail making test (TMT), frontal assessment battery (FAB), headache-related disability (MIDAS) and headache parameters. Then, participants were randomized into three groups: active exercise only (n = 10), cognitive task only (n = 10) and dual task protocol (n = 10). After 3 months of each treatment and after 1-month follow-up the same neurophysiological and clinical outcomes were revaluated. A significant time x group effect was only found for the trapezius muscle (p = 0.012, pη2 = 0.210), suggesting that PPT increased significantly only in active exercise and dual task protocol groups. A significant time effect was found for rMT (p < 0.001, pη2 = 0.473), MIDAS (p < 0.001, pη2 = 0.426), TMT (p < 0.001, pη2 = 0.338) and FAB (p < 0.001, pη2 = 0.462). A repeated measures ANOVA for SICI at 3 ms highlighted a statistically significant time effect for the dual task group (p < 0.001, pη2 = 0.629), but not for the active exercises group (p = 0.565, pη2 = 0.061), and for the cognitive training (p = 0.357, pη2 = 0.108). The dual task protocol seems to have a more evident effect on both habituation and sensitization outcomes than the two monotherapies taken alone in people with migraine.

## Introduction

Migraine represents the most common neurologic condition with an important socioeconomic burden, particularly for Italy, but also for many other countries. Part of migraine burden is due to its complex physiopathology that is still object of studies for a better understanding of the underlying mechanisms and, as a consequence, for a better choice of the related treatments and clinical management [1, 2]. Concerning physiopathology, researchers defined migraine “a brain state of altered excitability”[3] in a “migraine brain”. In fact, neurophysiological studies highlighted in people with migraine a general neuronal hyper-responsivity to innocuous sensory and noxious stimuli [4, 5], especially between attacks. This phenomenon may depend on two opposing processes: lack of habituation and sensitization that determine alteration in the sensory processing[3–8]. Habituation represents an inhibitory response to sensory stimulation. Sensitization represents an augmentation response to sensory stimulation[5]. On one hand, lack of habituation is clinically manifested by the alteration of cognitive processing, neurophysiologically it is displayed by an increase in cortical excitability and by a reduction of intracortical inhibition[6, 9–11]. On the other hand, sensitization is clinically manifested by an increase in headache parameters, neurophysiologically is displayed by an alteration of the pressure pain threshold over trigeminal and extra-trigeminal area[5, 12–15]. Taken together, lack of habituation and sensitization leads to an imbalance between excitatory-inhibitory transmission[16, 17], which in turn, results in cognitive impairment[8, 18, 19] and dysfunction in pain modulation [5, 20, 21].

Recent studies support the efficacy of non-pharmacological treatments in the management of migraine[12, 22, 23]. In the context of existing treatments for migraine, any treatments may act on sensitization pain modulation other on habituation: active exercise may result in neuroplasticity, through the release of the brain-derived neurotrophic factor, and in pain modulation, through the activation of endogenous systems[24–28]; cognitive training may result in neuroplasticity and global cognition, through the enhance of the performance on cognitive functions[29–31]. However, it seems that there are no treatments that may act both on sensitization and on habituation in people with migraine. Currently, the combination of active exercise with cognitive training in a concomitant dual task protocol represents an emerging treatment to strength their efficacy. Cognitively, a dual task protocol promotes divided attention, such as the ability to perform two or more tasks simultaneously, and executive functions, such as working memory, inhibition and shifting; physically, dual task protocol promotes gait, balance [32–37]. Moreover, it seems that the neuroplasticity induced by active exercise is further enhanced in dual task protocol, through an up-regulation of cortical connectivity[38]. These concomitant combination in a dual task protocol could act on both habituation and sensitization outcomes in migraine. To date, no previous study has investigated the efficacy of a dual task protocol in people with migraine. Furthermore, no previous study has compared the effect of different types of treatments on neurophysiological and clinical outcomes in people with migraine.

Based on previous literature [32–37], it could be hypothesized that a dual task protocol could be more effective than active exercise and cognitive training alone by combining the mechanisms of action on habituation and sensitization. Therefore, the first aim of this study was to investigate the efficacy of a dual-task protocol in people with episodic migraine with respect to both active exercises only and to cognitive training-only treatments, concerning some peripheral and central neurophysiological outcomes. The second aim was to evaluate the efficacy of a dual-task protocol in people with episodic migraine with respect to the two monotherapies concerning some clinical outcomes.

## Methods

A randomized control trial was adopted in people with episodic migraine without aura (ICHD-3)[39]. The project was approved by the institutional review board (CEUR 2021-Sper-26; ID 3672) and it was registered on ClinicalTrials.gov (identifier: NCT05596058). The informed consent was written by all participants. The first evaluation and enrolment were performed by a tertiary Headache Centre. The following inclusion criteria were respected: Episodic high-frequency migraine diagnosis (ICHD-3)[39]; Age between 18 and 65 years. Exclusion criteria were: migraine with aura; contraindications or low tolerance to TMS; other neurological or psychiatric disorders; cardiac implantable devices; current drug intake that may change the cortical excitability; previous migraine prophylaxis treatment in the last three months; comorbidities such as depression, anxiety, sleep disorders; participants that do not provide their consent to the study. Each participant received a diary to record the frequency of migraine, duration of the attack, migraine intensity and drug intake per month. After one month, each patient was evaluated, and the eligibility criteria were frequency of migraine from ≥ 8 to ≤ 14 days per month, according to diagnostic criteria of ICDH3-beta [39]. The enrolment began on the first of June 2022, the primary completion was performed on the first October 2022, the data collection was completed on the first November 2023 due to the unavailability of the subjects who give the consent to participate to the study protocol.

### Outcomes assessment

**Fig. 1:**
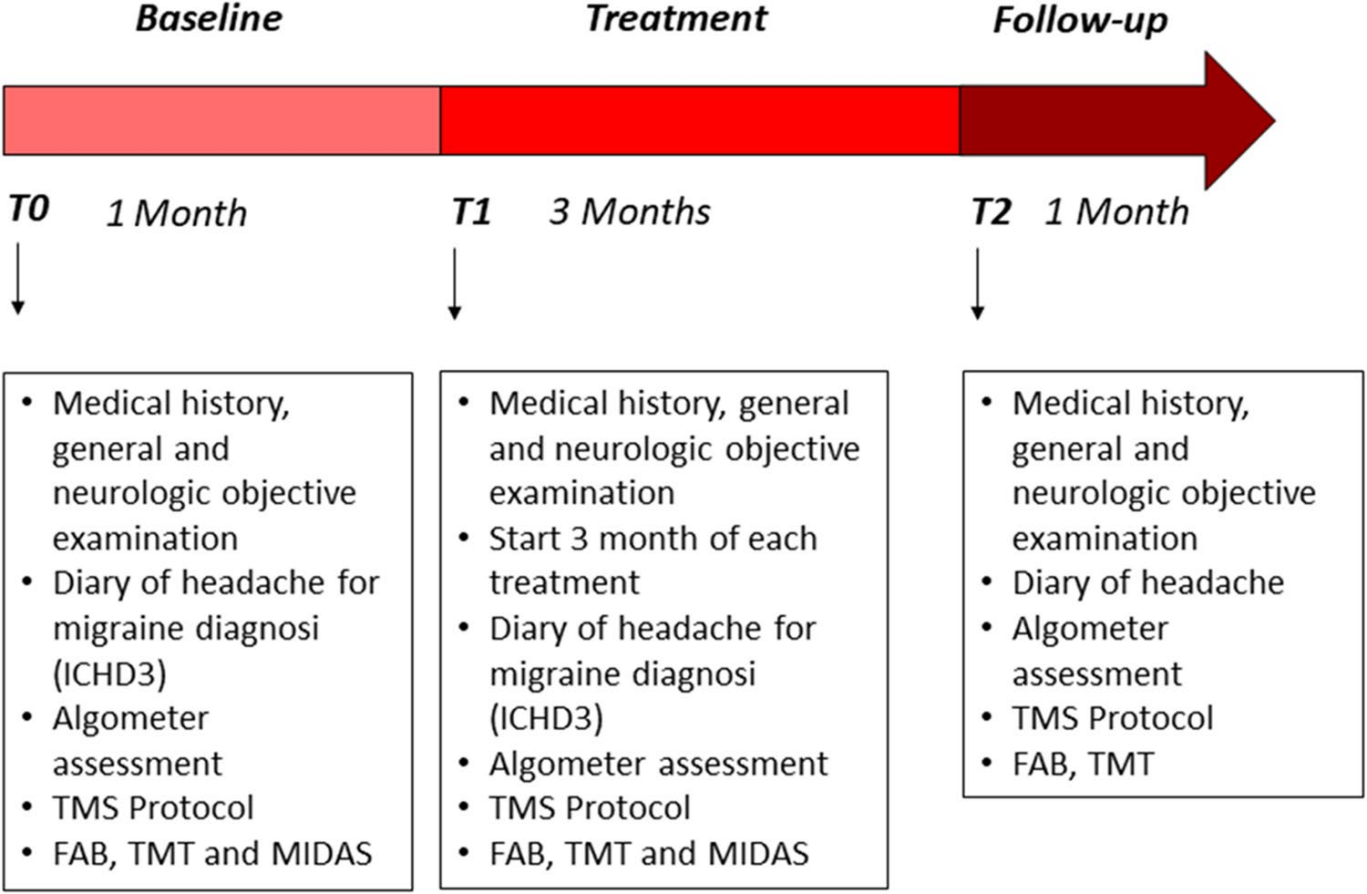
Time line of the study

Figure 1 presents an overview of the timeline of the study (Fig. 1).

#### Pressure pain threshold (PPT) assessment

PPT assessment was conducted according to previous guidelines[40]. Five muscles were chosen over the trigeminocervical complex (masseter, temporalis, procerus, trapezius and sub-occipitalis), and one muscle was chosen outside the trigemino-cervical (tensor fascia latae). Participants were asked to press the stop button when the pressure was felt as painful[22, 40]. The first pressure was applied on the wrist of each participant, in order to familiarize with the procedure[22, 40]. Three evaluations were carried out for each muscle with one-minute of interval between each evaluation. A rate of approximately 30 kPa/s was increased in each assessment [12, 40].

#### Transcranial magnetic stimulation (TMS)

The single-pulse protocol (sp) of TMS was used to teste the resting motor threshold (rMT) while the paired pulse protocol (pp) of TMS was used to assess the short interval intracortical inhibition (SICI) and the intracortical facilitation (ICF) over the left primary motor cortex (M1)[41–43]. A MagPro® magnetic stimulator (MagVenture Inc., Alpharetta, GA, USA) was used by connecting it to an electromyographic device (Synergy®, Natus®, Middleton, WI, USA). The amplitude of MEPs was calculated peak-to-peak for each evaluation[21]. The TMS parameters were collected in this order: 1) From Single-Pulse (sp)-TMS, the Resting Motor Threshold (rMT): the minimum stimulation intensity required to produce a peak-to-peak motor evoked potential (MEP) amplitude of ≥ 50 μV in at least 50% of five of ten consecutive stimuli[21, 42]; 2) From Paired Pulse (pp)-TMS, short interval intracortical inhibition (SICI): evoked by delivering a subthreshold (80% rMT) conditioning stimulus followed by a suprathreshold (130% rMT) test stimulus at interstimulus intervals (ISIs) of 3 and 5 ms. Four MEPs were recorded; 3) From Paired Pulse (pp)-TMS, intracortical facilitation (ICF): evoked by delivering a subthreshold (80% rMT) conditioning stimulus followed by a suprathreshold (130% rMT) test stimulus at ISIs of 10, 15 and 20 ms. Four MEPs were recorded [21, 44].

#### Executive functions

The Trail Making Test (TMT) and the Frontal Assessment Battery (FAB) were used to evaluate the executive functions[8, 45, 46]. TMT is divided into TMT A and TMT B[8, 45–47]. FAB explores 6 functions: conceptualization, mental flexibility, motor programming, sensitivity to interference, inhibitory control, and environmental autonomy. To reduce the risk of learning effect all participants were instructed to perform the executive functions tests at least 8 times in the week preceding the baseline evaluation.

#### Migraine Disability Assessment Scale (MIDAS)

The MIDAS consists of 5 questions concerning the following item [48]: absence from work or school, inability to carry out household chores, and to take part in family, social, or leisure activities.

#### Headache diary

From t0, the headache diary was used by participants during the 3 months of each treatment (t1) and after 1 month follow up (t2) to collect the following information: frequency of migraine; duration of attacks; pain intensity; symptomatic drugs intake; association with menstrual cycle[49]. The consumption of symptomatic medication was allowed maximum twice a week[50]. Participants that exceeded this frequency were considered dropouts in the final data analysis.

All the assessments were performed for all participants exclusively in the inter-ictal phase[51, 52]. In addition, participants had to not take any medication that may modify the cortical excitability for at least 72 h before the measurements[53]. In the case of female subjects, the evaluations were applied always in the late follicular phase [54].

After the baseline data collection (t0), participants were randomized into three groups with a stratification for age and sex (1:1:1): active exercise only, cognitive task only and dual-task protocol. One of the investigators who was not involved in outcomes assessment and physiotherapy protocols evaluated inclusion and exclusion criteria and randomized the subjects into the three groups. The examiner who performed the outcomes measure and the statistical analysis was blinded to the participants allocation. Also, participants were blinded concerning the other groups of treatments (Fig. 1).

### Intervention

#### Active exercise-only protocol

Active exercise-only protocol was scheduled in 20 one-hour individual sessions twice per week. The protocol was based on the review of Lemmens[26]. Each training session started with a 20-min warm-up by walking on a treadmill with a progressive increase in speed every 5 min. This part was followed by 30 min of strengthening exercises for the total body. Finally, in the last 10 min stretching exercises were applied with slow breathing[26]. The circuit of the exercise was changed every two weeks, with a total of 5 graded circuits during the three months of treatment (see supplementary material).

#### Cognitive training only protocol

The cognitive training-only protocol was scheduled in a total of 20 one-hour individual sessions twice per week. The protocol was based on previous studies for cognitive impairment[8, 31–36] and for migraine[29]. The goal was to engage the three core executive functions: inhibition, working memory and shifting[55]. In order to increase the difficulty, the cognitive task was constantly changed. A total of 15 min was spent on the inhibition goal: GO-NO-GO tasks (5 min), Stroop tasks (5 min) and inhibition of the correct answer (5 min). During the GO-NO-GO task, participants had to provide different responses to alternating signals i.e., “not tapping when the examiner taps twice and copying the examiner when he taps once”. In the Stroop task, participants had to name the name of a color (e.g., "blue", "green", or "red") printed in a different color. In the inhibition of the correct answer task, participants were asked to give the wrong answer to simple general culture questions presented and to inhibit the impulse to answer correctly. The working memory goal lasted 15 min. Several memory tasks were presented: counting down, word list recall, spelling a five-letter word backwards, subtracting by sixes or sevens from a randomly presented two-digit number, reciting the months in the reverse order starting from a randomly chosen month. The shifting goals lasted 15 min: mental flexibility tasks (5 min), Simon tasks (5 min) and Wisconsin Card Sorting Test (5 min). In mental flexibility tasks, patients listed as many words as they could in one minute. Word categories included words beginning with a specific letter or belonging to a specific semantic category or words that were in rhyme with a word chosen by the therapist. In the Simon task a blue or red square was presented on the left or the right side of the computer screen. The participants were asked to raise their left hand if the square was blue and the right hand if the square was red, irrespectively of the location of the square. The trials were either congruent or incongruent, where in the congruent trials, the stimulus location was on the same side as the response key, and in the incongruent trials on the opposite side. In the Wisconsin Card Sorting Test, 4 cards were presented to the patients, with numbers (from 1 to 4), colors (red, green, yellow, blue) and symbols (triangle, square, circle, star). A fifth card was then shown depicting one item from each of the four categories. The patients were asked to select the card among the first 4 presented that matched the fifth card. The software randomly changed the categories after a few repetitions (see supplementary material).

#### Dual-task protocol

The dual-task protocol was an integrated protocol of active exercise-only protocol with concomitant cognitive training-only protocol. A total of 20 one-hour individual sessions were conducted twice per week. Participants had to perform different cognitive tasks during concomitant active exercise scheduled in 5 graded circuits[32] (see supplementary material).

After 3 months of each treatment, participants were revaluated (t1) with the same neurophysiological and clinical outcomes (PPT; TMS; TMT; FAB; MIDAS; headache diary) and after 1-month follow-up with the same outcomes (PPT; TMS; TMT; FAB; MIDAS; headache diary) (Fig. 1).

### Statistical analyses

This is the primary analysis of this data. G*Power was used to demine posteriori the achieve power considering the main finding of this study, being > 0.95. The statistical analyses were performed with SPSS version 23 (IBM). Data are reported as means, standard deviations (SDs), and 95% confidence intervals (CI), or counts and proportions (%) as appropriate. Two-tailed testing was performed. To evaluate the effect of the different treatment protocols on the assessed outcomes, a mixed-factors analysis of variance (ANOVA) was performed for the different measures (within-groups: time – t0, t1, t2; between-groups: treatment—dual tasks, active exercise, cognitive training), considering also the possible effect of interstimulus interval for TMS (SICI, within-groups: ISI—3 ms, 5 ms; ICF, within-groups: ISI—10 ms, 15 ms, 20 ms), and the possible effect of the side of the body for PPT (within-groups: side—right, left). In case of significant main effects, a postdoc analysis with Sidak's correction was performed. Due to the large variability within groups for SICI at 3 ms, a repeated measures ANOVA was also performed separately for each group. Normality testing was performed by using the Shapiro–Wilk test was on all the datasets. Significance was set at p < 0.05.

## Results

**Fig. 2:**
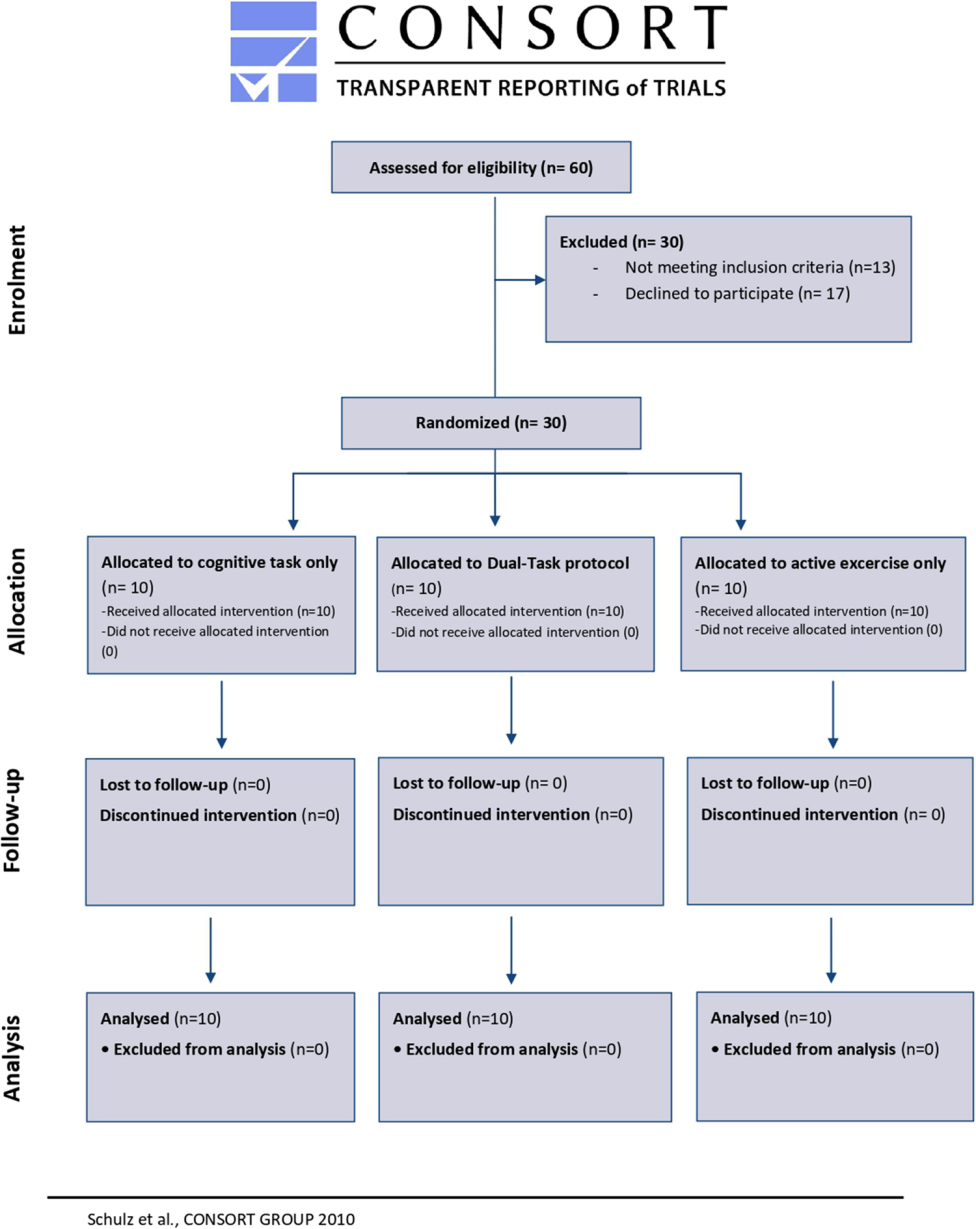
Consort flow chart diagram for trial

A total of 30 adults with episodic high-frequency migraine were included (Fig. 2)[56]. The active exercise-only group consisted of 2 men and 8 females (36.5 ± 13.9); the cognitive task-only group consisted of 2 men and 8 females (mean age 42.7 ± 11.2); the dual task protocol group consisted of 2 men and 8 females (mean age 48.3 ± 9.7). There was no missing data.

### Pressure Pain Threshold

No time x group x side, time x side, nor group x side effects were observed for any of the assessed muscles. A significant time x group effect was only found for the trapezius muscle (F_4,54_ = 3.588, p = 0.012, pη2 = 0.210). A significant time effect was found for the procerus muscle (F_2,54_ = 3.472, p = 0.038, pη2 = 0.114), but not for the other assessed muscles. In addition, a significant side effect was only found for the trapezius muscle (F_1,27_ = 12.719, p = 0.001, pη2 = 0.320). In particular, the PPT over the trapezius muscle was suggested to increase over time in the dual task group and in the active exercises group, whereas in the cognitive training group the PPT decreased over time (Table 1). Regarding the procerus muscle, the pain threshold did not significantly increase between t0 and t1 (28.2 kPa; 95% CI: -15.1—71.4; p = 0.293), between t1 and t2 (17.6 kPa; 95% CI: -12.1—47.3.5; p = 0.369), and between t0 and t2 (45.7 kPa; 95% CI: -11.0—102.5; p = 0.142).

**Table 1:** Pressure pain threshold in participants with migraine before (t0), after 3 months (t1) and at 1-month follow-up (t2), divided according to the protocol: the dual-task protocol (n = 10), active exercise only protocol (n = 10), cognitive training only protocol (n = 10). Data are shown as mean ± standard deviation

	Dual-task (n = 10)		Active exercise (n = 10)		Cognitive training (n = 10)	
	*Left*	*Right*	*Left*	*Right*	*Left*	*Right*
*Masseter*						
Pre (t0)	176.9 ± 25.6	195.8 ± 25.5	216.2 ± 25.6	224.7 ± 25.5	215.5 ± 25.6	209.9 ± 25.5
Post (t1)	204.8 ± 26.5	210.5 ± 26.0	219.3 ± 26.5	253.4 ± 26.0	172.0 ± 26.5	189.6 ± 26.0
follow-up (t2)	231.6 ± 26.7	235.0 ± 23.8	222.7 ± 26.7	226.2 ± 23.8	189.0 ± 26.7	182.8 ± 23.8
*Suboccipital*						
Pre (t0)	283.9 ± 53.5	269.1 ± 43.4	358.0 ± 53.5	333.0 ± 43.4	328.9 ± 53.5	329.0 ± 43.4
Post (t1)	378.3 ± 41.1	327.5 ± 42.4	361.1 ± 41.1	325.2 ± 42.4	283.2 ± 41.1	303.6 ± 42.4
follow-up (t2)	390.9 ± 43.7	358.2 ± 45.3	330.5 ± 43.7	343.3 ± 45.3	296.3 ± 43.7	295.7 ± 45.3
*Temporalis*						
Pre (t0)	216.6 ± 34.9	244.9 ± 33.0	274.4 ± 34.9	288.8 ± 33.0	286.9 ± 34.9	278.4 ± 33.0
Post (t1)	289.2 ± 44.4	295.4 ± 42.6	310.8 ± 44.4	299.2 ± 42.6	273.1 ± 44.4	264.1 ± 42.6
follow-up (t2)	319.5 ± 40.2	323.5 ± 39.3	324.4 ± 40.2	323.6 ± 39.3	272.4 ± 40.2	239.0 ± 39.3
*Trapezius*						
Pre (t0)	319.2 ± 50.1	377.5 ± 50.8	323.4 ± 50.1	411.2 ± 50.8	357.7 ± 50.1	394.2 ± 50.8
Post (t1)	405.5 ± 43.8	436.9 ± 50.4	361.4 ± 43.8	404.7 ± 50.4	297.7 ± 43.8	304.4 ± 50.4
follow up (t2)	469.6 ± 49.1	539.9 ± 48.8	378.0 ± 49.1	405.5 ± 48.8	292.8 ± 49.1	288.2 ± 48.8
*TFL*						
Pre (t0)	554.0 ± 80.6	628.0 ± 87.2	486.2 ± 80.6	579.9 ± 87.2	509.3 ± 80.6	502.2 ± 87.2
Post (t1)	661.7 ± 95.9	629.3 ± 94.7	567.1 ± 95.9	576.6 ± 94.7	537.1 ± 95.9	521.0 ± 94.7
follow up (t2)	645.6 ± 88.4	771.6 ± 110.4	576.0 ± 88.4	613.5 ± 110.4	479.3 ± 88.4	483.1 ± 110.4
*Procerus*						
Pre (t0)	234.8 ± 34.8		280.6 ± 34.8		234.1 ± 34.8	
Post (t1)	299.0 ± 33.0		272.2 ± 33.0		262.8 ± 33.0	
follow up (t2)	294.8 ± 38.4		318.3 ± 38.4		273.7 ± 38.4	

Masseter: Masseter muscles’ pain threshold (kPa); Suboccipital: Suboccipital muscles’ pain threshold (kPa); Temporalis: Temporalis muscles’ pain threshold (kPa); Trapezius: Trapezius muscles’ pain threshold (kPa); TFL: Tensor Fascia Latae muscles’ pain threshold (kPa); Procerus muscles’ pain threshold (kPa).

### Transcranial Magnetic Stimulation (TMS)

#### Single-pulse TMS Protocol

No time x group effect (F_4,54_ = 0.501, p = 0.735, pη2 = 0.036) and no group effect (F_2,27_ = 0.443, p = 0.647, pη2 = 0.032) were found for rMT, while a statistically significant time effect was observed (F_2,54_ = 24.208, p < 0.001, pη2 = 0.473) (Table 2). In fact, it significantly increased from t0 to t1 (13.8%; 95% CI: 7.1–20.5; p < 0.001), and between t0 and t2 (15.6%; 95% CI: 8.2–23.1; p < 0.001), but not from t1 to t2 (1.8%; 95% CI: -2.3—6.0; p = 0.608).

**Table 2:** TMS outcomes before, after 3 months and at 1-month follow-up, divided according to the protocol: dual-task protocol group (n = 10) active exercise only protocol group (n = 10) or cognitive exercise only protocol group (n = 10). Data are represented as mean ± standard deviation

	Dual-task (n = 10)	Active exercise (n = 10)	Cognitive training (n = 10)
*SICI—ISI 3 ms (mV)*			
Pre (t0)	0.200 ± 0.082	0.270 ± 0.082	0.150 ± 0.082
Post (t1)	0.051 ± 0.104	0.269 ± 0.104	0.231 ± 0.104
Follow-up (t2)	0.062 ± 0.027	0.120 ± 0.027	0.071 ± 0.027
*SICI—ISI 5 ms (mV)*			
Pre (t0)	0.606 ± 0.173	0.270 ± 0.173	0.272 ± 0.173
Post (t1)	0.309 ± 0.094	0.378 ± 0.094	0.156 ± 0.094
Follow-up (t2)	0.410 ± 0.191	0.227 ± 0.191	0.333 ± 0.191
*ICF—ISI 10 ms (mV)*			
Pre (t0)	0.866 ± 0.222	0.700 ± 0.222	0.391 ± 0.222
Post (t1)	0.689 ± 0.147	0.520 ± 0.147	0.328 ± 0.147
Follow-up (t2)	0.828 ± 0.289	0.860 ± 0.289	0.445 ± 0.289
*ICF—ISI 15 ms (mV)*			
Pre (t0)	0.785 ± 0.184	0.630 ± 0.184	0.380 ± 0.184
Post (t1)	0.670 ± 0.190	0.610 ± 0.190	0.349 ± 0.190
Follow up (t2)	1.103 ± 0.395	1.260 ± 0.395	0.780 ± 0.395
*ICF—ISI 20 ms (mV)*			
Pre (t0)	0.870 ± 0.210	0.540 ± 0.210	0.345 ± 0.210
Post (t1)	0.880 ± 0.244	0.660 ± 0.244	0.364 ± 0.244
Follow up (t2)	1.015 ± 0.390	1.250 ± 0.390	0.944 ± 0.390
*rMT (% SO)*			
Pre (t0)	55.5 ± 3.7	56.6 ± 3.7	56.5 ± 3.7
Post (t1)	71.6 ± 4.1	66.1 ± 4.1	72.3 ± 4.1
Follow up (t2)	72.1 ± 4.3	68.4 ± 4.3	75.0 ± 4.3

*SICI* Short Interval Cortical Inhibition, *ISI* Interstimulus interval, *ICF* Intracortical Facilitation, *rMT* Resting motor threshold, *SO* Stimulator output

#### Paired-pulse TMS protocol

Concerning SICI, only a significant ISI effect was found (F_1,27_ = 6.896, p = 0.014, pη^2^ = 0.203). In fact, the MEP value was significantly higher at 5 ms than 3 ms (0.171 mV; 95% CI: 0.037–0.304; p = 0.014). A repeated measures.

**Fig. 3:**
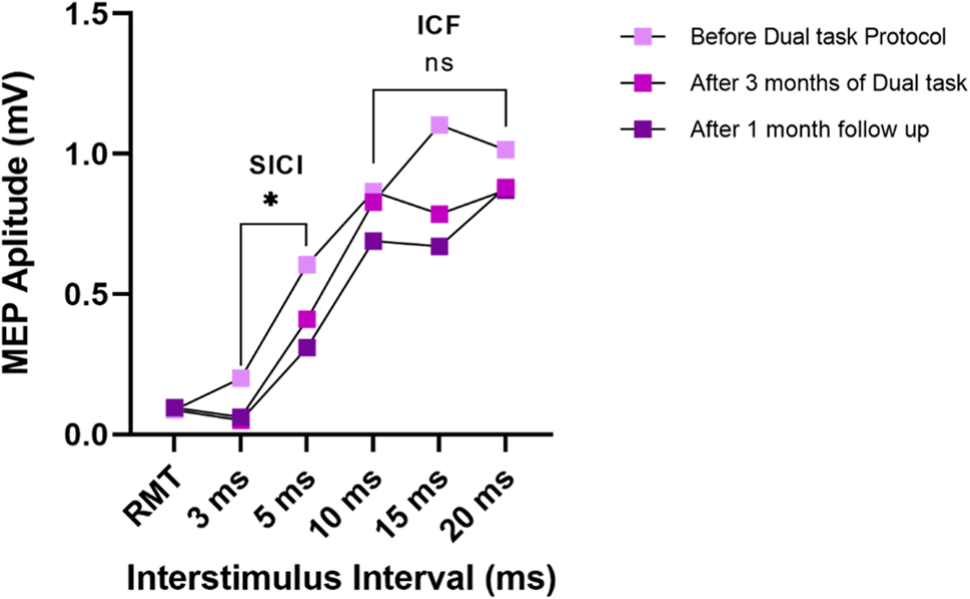
Transcranial Magnetic Stimulation pared pulse protocol. Transcranial magnetic stimulation (TMS) motor evoked potentials (MEPs) at different interstimulus intervals (ISI) of participants with migraine (n=10) before the dual task protocol (t0) after 3 months of dual task protocol (t1) and after 1-month follow-up (t2)

ANOVA for SICI at 3 ms highlighted a statistically significant time effect for the dual task group (F_2,18_ = 15.251,

p < 0.001, pη^2^ = 0.629), but not for the active exercises group (F_2,18_ = 0.589, p = 0.565, pη^2^ = 0.061), and for the cognitive training (F_2,18_ = 1.090, p = 0.357, pη^2^ = 0.108). In fact, for the dual task group the MEP value at 3 ms significantly decreased between t0 and t1 (-0.149 mV; 95% CI: -0.230—-0.068; p = 0.001), and from t0 to t2 (0.138 mV; 95% CI: -0.247—-0.029; p = 0.015), but not between t1 and t2 (0.011 mV; 95% CI: -0.058—0.080 p = 0.956). In the active exercises group, the MEP value at 3 ms did not significantly decrease from t0 to t1 (-0.001 mV; 95% CI: -0.582—0.580; p > 0.999), between t0 and t2 (-0.150 mV; 95% CI: -0.509—0.209; p = 0.583), and from t1 to t2 (-0.149 mV; 95% CI: -0.576—0.278; p = 0.705). In the cognitive training group, the MEP value at 3 ms significantly decreased between t0 and t2 (-0.079 mV; 95% CI: -0.151—-0.008; p = 0.030), but not from t0 to t1 (-0.081 mV; 95% CI: -0.307—0.470; p = 0.913), and not between t1 and t2 (-0.160 mV; 95% CI: -0.543—0.222; p = 0.581) (Fig. 3). Concerning ICF, only a significant time x ISI effect was observed (F_4,108_ = 2.909, p = 0.048, pη2 = 0.097) (Table 2).

### Cognitive functions

A significant time effect was observed for TMT (F_2,54_ = 13.812, p < 0.001, pη2 = 0.338), which significantly improved between t0 and t1 (-6.2 s, 95% CI: -10.5—-1.8, p = 0.004), and from t0 to t2 (-9.2 s, 95% CI: -14.1—-4.3, p < 0.001), whereas it was not statistically significant between t1 and t2 (- 3.1 s, 95% CI: -7.5 –1.3, p = 0.238). A significant time effect was found in the FAB assessment (F_2,54_ = 23.173, p < 0.001, pη2 = 0.462), showing a significant improvement between t0 and t1 (1.1, 95% CI: 0.5 – 1.8, p < 0.001), and from t0 to t2 (1.3, 95% CI: 0.7 – 1.8, p < 0.001), whereas it was not statistically significant between t1 and t2 (0.1, 95% CI: -0.2 –0.5, p = 0.707) (Table 3).

**Table 3:** Cognitive assessment in participants with migraine before, after 3 months, and at 1-month follow-up, divided according to the protocol: dual-task protocol group (n = 10), active exercise only protocol group (n = 10), and cognitive training only protocol group (n = 10). Data are reported as mean ± standard deviation

	Dual-task (n = 10)	Active exercise (n = 10)	Cognitive training (n = 10)
*TMT*			
Pre (t0)	34.2 ± 4.9	20.0 ± 4.9	28.3 ± 4.9
Post (t1)	26.6 ± 3.8	18.5 ± 3.8	19.0 ± 3.8
Follow-up (t2)	22.1 ± 3.3	17.4 ± 3.3	15.4 ± 3.3
*FAB*			
Pre (t0)	16.3 ± 0.4	16.6 ± 0.4	16.8 ± 0.4
Post (t1)	17.7 ± 0.2	17.8 ± 0.2	17.6 ± 0.2
Follow-up (t2)	17.8 ± 0.2	17.8 ± 0.2	17.9 ± 0.2

*TMT* Trail Making Test, *FAB* Frontal Assessment Battery

### MIDAS

No time x group effect (F_2,27_ = 0.264, p = 0,770, pη2 = 0.019), nor no group effect were observed (F_2,27_ = 0.255, p = 0.776, pη2 = 0.019), while a statistically significant time effect was found (F_1,27_ = 20.006, p < 0.001, pη2 = 0.426). Indeed, the MIDAS scores significantly decreased from t0 to t1 (-26.9; 95% CI: -39.2—-14.6; p < 0.001) (Table 4).

**Table 4:** Headache Diary for participants with migraine before, after 3 months, and at 1-month follow-up, divided according to the protocol: dual-task protocol group (n = 10), active exercise only protocol group (n = 10), cognitive training only protocol group (n = 10). Data are reported as mean ± standard deviation

	Dual-task (n = 10)	Active exercise (n = 10)	Cognitive training (n = 10)
*Frequency*			
Pre (t0)	11.0 ± 1.5	11.7 ± 1.5	9.9 ± 1.5
Post (t1)	7.7 ± 1.9	9.6 ± 1.9	10.0 ± 1.9
Follow-up (t2)	6.8 ± 1.5	9.5 ± 1.5	8.5 ± 1.5
*Duration*			
Pre (t0)	87.8 ± 17.4	83.3 ± 17.4	46.9 ± 17.4
Post (t1)	47.7 ± 14.8	66.7 ± 14.8	48.2 ± 14.8
Follow-up (t2)	49.9 ± 15.0	64.8 ± 15.0	48.1 ± 15.0
*Low intensity*			
Pre (t0)	31.0 ± 8.1	25.5 ± 8.1	12.2 ± 8.1
Post (t1)	26.0 ± 9.0	26.8 ± 9.0	15.2 ± 9.0
Follow-up (t2)	28.0 ± 9.5	28.8 ± 9.5	12.2 ± 9.5
*Medium intensity*			
Pre (t0)	23.8 ± 6.7	32.6 ± 6.7	18.8 ± 6.7
Post (t1)	15.2 ± 6.3	26.3 ± 6.3	21.5 ± 6.3
Follow up (t2)	11.6 ± 6.9	21.6 ± 6.9	25.5 ± 6.9
*High intensity*			
Pre (t0)	33.0 ± 12.6	25.5 ± 12.6	15.9 ± 12.6
Post (t1)	6.5 ± 3.7	13.9 ± 3.7	11.5 ± 3.7
Follow up (t2)	10.8 ± 5.1	16.2 ± 5.1	10.4 ± 5.1
*Drugs intake*			
Pre (t0)	12.7 ± 2.9	9.9 ± 2.9	10.4 ± 2.9
Post (t1)	6.1 ± 2.2	6.7 ± 2.2	8.5 ± 2.2
Follow up (t2)	5.8 ± 2.2	6.3 ± 2.2	9.0 ± 2.2
*MIDAS*			
	47.7 ± 40.9	47.9 ± 26.3	50.6 ± 37.3
	16.5 ± 14.4	27.0 ± 23.2	27.7 ± 47.1

Frequency: Monthly Headache Days; Duration: Monthly Headache Hours; Low intensity: Monthly hours of low intensity pain; Medium intensity: Monthly hours of medium intensity pain; High intensity: Monthly hours of high intensity pain; Drugs intake: Monthly headache drugs intake. MIDAS: migraine disability assessment test. Only assessed before and after the treatment per standardized protocols

### Headache diary

No significant time x group nor group effects were observed for the assessed parameters. No time effects were found about low-intensity pain (F_2,54_ = 0.018, p = 0.982, pη2 = 0.001) and medium-intensity pain (F_2,54_ = 1.664, p = 0.199, pη2 = 0.058), while a statistically significant effect was observed for frequency (F_2,54_ = 6.461, p = 0.003, pη2 = 0.193), duration of attack (F_2,54_ = 4.443, p = 0.016, pη2 = 0.141), high-intensity pain (F_2,54_ = 3.738, p = 0.030, pη2 = 0.122), and drugs intake (F_2,54_ = 8.652, p = 0.001, pη2 = 0.243). Frequency significantly decreased from t0 to t2 (-2.6; 95% CI: -4.4—-0.8; p = 0.003), but not between t0 and t1 (-1.8; 95% CI: -4.0—0.4; p = 0.142), and not from t1 to t2 (-0.8; 95% CI: -2.5 – 0.8; p = 0.488). Drugs intake significantly decreased between t0 and t1 (-3.9; 95% CI: -6.8—-1.0; p = 0.007), and from t0 to t2 (-4.0; 95% CI: -7.3—-0.7; p = 0.014), but not between t1 and t2 (-0.1; 95% CI: -2.0—1.9; p > 0.999) (Table 4).

## Discussion

The present study has evaluated, for the first time, the efficacy of a dual task protocol, i.e., active exercise plus cognitive task, on neurophysiological and clinical outcomes related to sensitization and habituation in people with migraine. The first interesting finding was that the pressure pain threshold (PPT) increased only in active exercise and dual-task protocol groups, suggesting a more positive effect on sensitization. On the other side, resting motor threshold, migraine-related disability, and neurophysiological tests improved, without differences, among all groups. Finally, the most remarkable finding was that short intracortical inhibition seems to be normalized only by the dual task protocol, suggesting a more effective action on the lack of habituation. Furthermore, the dual task protocol reported a higher migraine responders’ rate, suggesting a more effective action also on sensitization.

With regard to PPT, the findings supported the idea that active exercise could have a desensitization effect. Although no differences were reported between the active exercise only and dual-task group, the dual task seems to be more useful: the dual task protocol increased the PPT over 11 out of 11 muscles at both t1 and t2; the active exercise increased the PPT over 8 out of 11 muscles at t1, and over 9 out of 11 at t2. On the other side, the cognitive training only seems not to change the PPT: in fact, the PPT increased over only 3 out of 11 muscles at both t1 and over 1 of 11 at t2. No previous study had investigated the PPT variation after a dual-task protocol. Even though, previous research on people with chronic migraine showed a reduction of the PPT over all the muscles after an integrated protocol of active exercise and manual therapy[12]. This result supported the peripheral and central desensitization action of active exercise[24, 25, 28]: peripherally through the suppression of pain signals in the spinal dorsal horn, centrally through the activation of the brain areas involved in pain modulation. Particularly for migraine, it seems that high-intensity exercise has a direct effect on calcitonin gene-related peptide (CGRP): the release of the endocannabinoid ligand anandamide (AEA) inhibits the vasodilatation of the dural induced by CGRP[57]. This, in turn, may lead to a reduction of the PPT. It can be suggested, therefore, that dual-task protocol with active exercise may act on sensitization in people with migraine.

Concerning rMT, it reflects the excitability of cortico-cortical axons[58]. The resting motor threshold (rMT) increased after the all the three treatments, without differences. This result supports that active exercise increases motor evocated potential (MEP) due to a neuroplasticity mechanism [59–62]. No previous research highlighted change in rMT after cognitive training, but previous research has highlighted that cognitive training can promote neuroplasticity and global cognition in cognitive impairment[29, 31, 32]. The increase of MEP in people with migraine after cognitive training may be related to the rise in the performance of executive functions, which, in turn, leads to an improved lack of habituation. Consequently, the association of active exercise with concomitant cognitive tasks in a dual-task protocol may be more effective on the neuroplasticity mechanism in the motor cortex. Despite the analysis of variance did not report significant interaction among groups, rMT was enhanced more by three months of the dual task protocol with respect to the two monotherapies. Therefore, it is possible to hypothesize that the dual task may reduce cortical excitability due to a lack of habituation.

SICI and ICF represent a neurophysiological outcome of the inhibitory circuits mediated by GABAergic neurotransmission (SICI) and excitatory mediated by glutamatergic neurotransmission (ICF), respectively [63]. Studies agree on a significant reduction of SICI in people with migraine, but the data regarding ICF are still not clear [11, 64–67]. Our finding, for the first time, suggests that the dual task protocol may normalize the inhibitory circuits mediated by the GABAergic neurotransmission (SICI). This normalization was not highlighted in the two monotherapies. In fact, the neuroplasticity induced by the two monotherapies seems to be strongly increased by their concomitant association: a dual-task protocol may up-regulate the cortical connectivity related to cognitive tasks through activity-dependent learning that strengthens the synapses associated with the cognitive tasks [38]. Therefore, this up-regulation may promote associative learning and neuroplasticity and, consequently, may reduce the lack of habituation.

As regards cognitive functions, all treatments improve cognitive performance without differences among groups. It seems that cognitive impairment in people with migraine is due to central sensitization, migraine comorbidity/disability and lack of habituation. Active exercise may act centrally, with the release of the brain-derived neurotrophic factor that promotes cognitive performance and neuroplasticity, and peripherally, with the reduction of central sensitization that restores cognitive functioning [24, 25, 28]. On the other hand, cognitive training directly enhances executive functions that may reduce migraine-related disability and lack of habituation. Finally, the cognitive performance obtained after the dual-task protocol may reflect an action of the lack of habituation: on the one side the divided attention is promoted by the performance of two or more tasks simultaneously, on the other side, associative learning is potentiated by the up-regulation of synapses related to the cognitive tasks[38].

Concerning MIDAS and headache parameters, no differences were highlighted among the three treatments. However, the dual task protocol seems to have a more evident effect with respect to the two monotherapies in migraine-related disability, duration of migraine, drug intake. Data suggest also a less evident reduction after cognitive training only of all headache parameters. In line with the literature, data confirm that active exercise may be more effective in the reduction of migraine frequency in respect to the reduction of pain intensity[26, 68]. It is possible that the dual-task protocol is more effective in the improvement of clinical parameters with respect to active exercise only and cognitive training only.

The present study presents some limitations; first of all, the small sample size could have impacted statistical power and therefore suggests caution when interpreting the statistical findings; in addition, it did not allow sex stratification. In fact, sex plays a role in pain modulation and cortical excitability. In order to limit this variable, the proportion of males and females was the same in all the tested groups. Second, the guidelines of the International Headache Society allow the consumption of symptomatic medication [50]. Therefore, the only possibility was to limit the symptomatic drug intake to twice a week and to exclude the final analysis of the participants that exceeded this limit, as in previous studies on this topic [50]. Third, a long-term follow up would be necessary to determine the sustainability of the interventions’effects over time. Third, a long-term follow up would be necessary to determine the sustainability of the interventions’ effects over time. Despite these limitations, the study presents some strong novel points: first, the efficacy of an emerging non-pharmacological treatment, i.e., dual-task protocol, was investigated for the first time in people with migraine; second, the neurophysiological effects of three non-pharmacological treatments were assessed for the first time in people with migraine; third, the clinical effects of three non-pharmacological treatments were compared for the first time in people with migraine.

## Conclusion

A dual task protocol, with concomitant cognitive training and active exercise, in people with migraine seems to be useful both in habituation and in sensitization outcomes. To evaluate the generalizability of our findings, future studies are encouraged on larger samples, including sex and age stratification. In addition, long-term follow up could assess the sustainability of the intervention’s effects over time and allow a deeper investigation of the differences among treatments. Finally, the effect of the combination of both pharmacological treatments and dual task protocols on habituation and sensitization outcomes is still to be investigated and could provide an added value for clinical translation of these findings. Since different migraine populations and migraine types might respond differently to the proposed interventions, future studies are encouraged to evaluate the effects of the dual task protocol in different migraine types, such as chronic migraine and migraine with aura. Finally, these potential mechanisms observed in the dual-task protocol should be investigated in other pathologies and also with other method such as blink reflex, Contingent negative variation and P300, auditory, visual and somatosensory evoked potential, Blood flow and oxygen consumption with functional neuroimaging [4].

## Data Availability

The principal author takes full responsibility for the data presented in this study, analysis of the data, conclusions, and conduct of the research. The datasets page containing authors’ details analyzed during the current study are available from the corresponding author on reasonable request.

## Abbreviations

PPT: Pressure pain threshold
TMS: Transcranial magnetic stimulation
rMT: Resting motor threshold
ISIs: Interstimulus intervals
Sp TMS: Single-pulse transcranial magnetic stimulation
PP TMS: Paired pulse transcranial magnetic stimulation
SICI: Short interval intracortical inhibition
ICF: Intracortical facilitation
FAB: Frontal assessment Battery
TMT: Trail making test
MIDAS: Migraine disability assessment scale
CGRP: Calcitonin gene-related peptide
MEP: Motor evocated potential
